# Multilocus sequence typing (MLST) of JP2 genotype isolates of *Aggregatibacter actinomycetemcomitans* collected from carriers of African and non-African origin

**DOI:** 10.1080/20002297.2017.1325268

**Published:** 2017-06-20

**Authors:** Rolf Claesson, Carola Höglund Åberg, Dorte Haubek, Anders Johansson

**Affiliations:** ^a^ Division of Molecular Periodontology and Oral Microbiology, Department of Odontology, Faculty of Medicine, Umeå University, Umeå, Sweden; ^b^ Dorte Haubek, Section for Pediatric Dentistry, Department of Dentistry and Oral HealthAarhus Universitet, Denmark

## Abstract

The bacterium *Aggregatibacter actinomycetemcomitans* is associated with aggressive periodontitis. Individuals colonised with the highly leukotoxic JP2 genotype of the bacterium, are at increased risk for developing periodontitis. The JP2 genotype is considered to emerge from North Africa and subsequently spread to individuals of African origin, living geographically widespread including in other parts of Africa and outside Africa. Reports of non-African carriers of the JP2 genotype are scares. However, in this study we characterize by multilocus sequence analysis JP2 genotype isolates collected from individuals of both African and non- African origin.

**Materials and Methods:** The study collection comprised 43 JP2 genotype strains. Among those 23 were isolated at the Clinical laboratory of Dental School, Umeå, Sweden, from samples collected from patients living in Sweden, but of both non-Africa and African origin. Seven housekeeping genes were sequenced and the strains were distributed according to different sequence types (ST).

**Results:** In total, 8 ST were identified. The 11 isolates collected from patients of non-African origin were distributed in two ST groups, while the 12 isolates from patients of African origin were distributed in eight ST groups.

**Conclusions:** The JP2 genotype colonizing individuals of African origin may be more susceptible to mutations than those colonizing non- African individuals.

